# Pre-emptive active drainage of reflux (PARD) in Ivor-Lewis oesophagectomy with negative pressure and simultaneous enteral nutrition using a double-lumen open-pore film drain (dOFD)

**DOI:** 10.1007/s00464-021-08933-w

**Published:** 2022-01-01

**Authors:** Gunnar Loske, Johannes Müller, Wolfgang Schulze, Burkhard Riefel, Christian Theodor Müller

**Affiliations:** Department for General, Abdominal, Thoracic and Vascular Surgery, Katholisches Marienkrankenhaus Hamburg gGmbH, Alfredstrasse 9, 22087 Hamburg, Germany

**Keywords:** Endoscopic vacuum therapy, Drainage, Prophylaxis, Anastomosis, Endoscopy, Nasogastric tube

## Abstract

**Background:**

Postoperative reflux can compromise anastomotic healing after Ivor-Lewis oesophagectomy (ILE). We report on Pre-emptive Active Reflux Drainage (PARD) using a new double-lumen open-pore film drain (dOFD) with negative pressure to protect the anastomosis.

**Methods:**

To prepare a dOFD, the gastric channel of a triluminal tube (Freka®Trelumina, Fresenius) is coated with a double-layered open-pore film (Suprasorb®CNP drainage film, Lohmann & Rauscher) over 25 cm. The ventilation channel is blocked. The filmcoated segment is placed in the stomach and the intestinal feeding tube in the duodenum. Negative pressure is applied with an electronic vacuum pump (− 125 mmHg, continuous suction) to the gastric channel. Depending on the findings in the endoscopic control, PARD will either be continued or terminated.

**Results:**

PARD was used in 24 patients with ILE and started intraoperatively. Healing was observed in all the anastomoses. The median duration of PARD was 8 days (range 4–21). In 10 of 24 patients (40%) there were issues with anastomotic healing which we defined as “at-risk anastomosis”. No additional endoscopic procedures or surgical revisions to the anastomoses were required.

**Conclusions:**

PARD with dOFD contributes to the protection of anastomosis after ILE. Negative pressure applied to the dOFD (a nasogastric tube) enables enteral nutrition to be delivered simultaneously with permanent evacuation and decompression.

**Supplementary Information:**

The online version contains supplementary material available at 10.1007/s00464-021-08933-w.

Postoperative reflux (PR) after Ivor-Lewis oesophagectomy (ILE) can have a deleterious effect on anastomotic wound healing and is a known risk of pulmonary aspiration. The main causes of PR are: paralysis, a pressure gradient between positive intraabdominal and negative intrathoracic pressure, and resection of the distal oesophageal sphincter. Until now PR has been drained using nasogastric tubes (NGT) [1–3]. Since common NGTs function in a passive manner using gravity and capillary force, drainage of PR remains incomplete, and the intrathoracic anastomosis becomes contaminated with digestive secretions [4].

We describe the novel method of a pre-emptive active reflux drainage (PARD) with continuous negative pressure using a new double-lumen open-pore film drainage (dOFD) in order to completely drain and decompress the gastric conduit with the anastomotic region [5, 6]. This novel type of a NGT enables permanent suction and enteral nutrition to be delivered simultaneously. We report here on our first experience with PARD after ILE in a primary observation series of 24 patients.

## Materials and methods

**Fig. 1:**
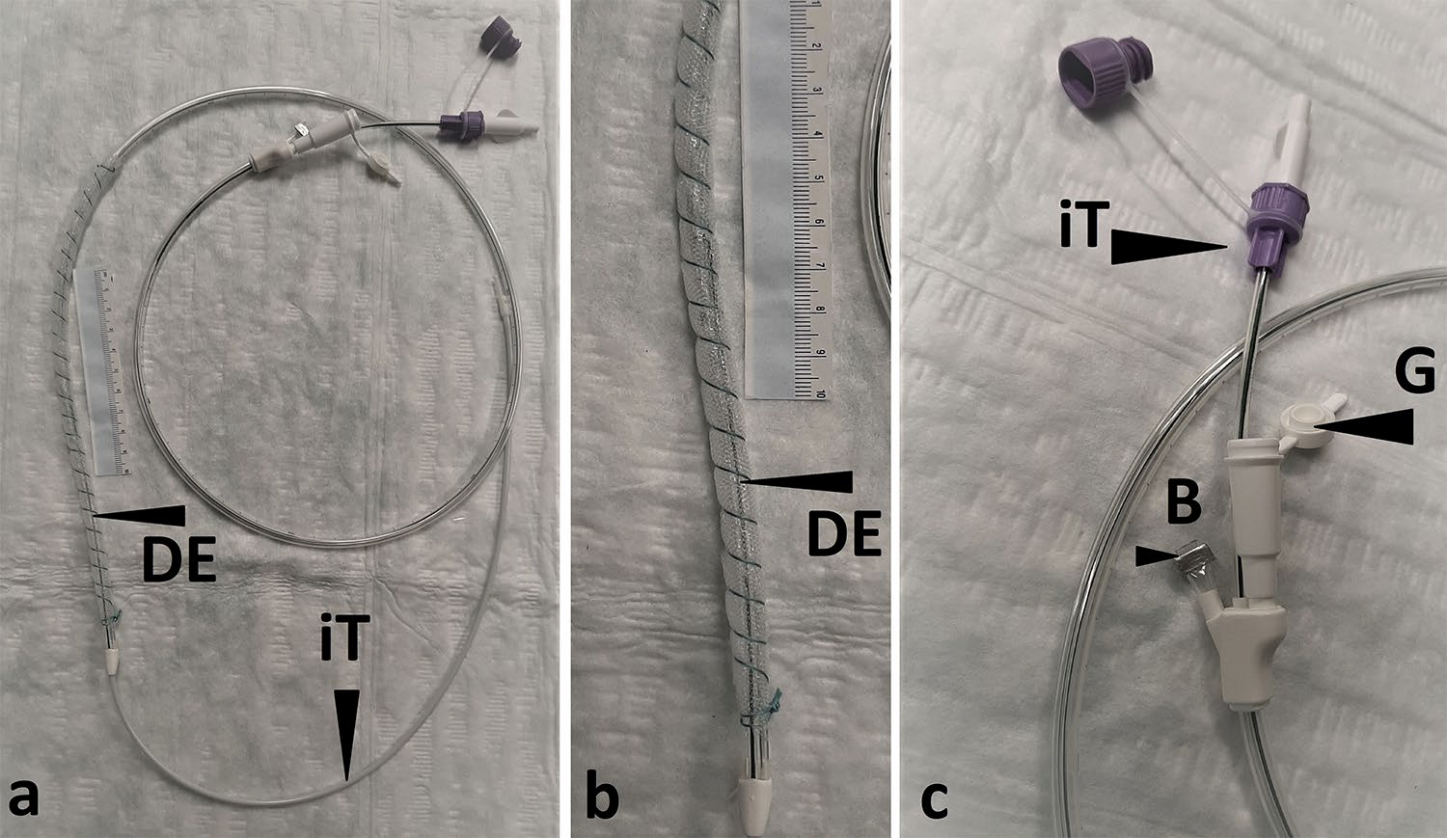
Showing the dOFD, **a** open-pore drainage-element (DE) coated with a 25 cm long strip of the open-pore double-layered thin film. Intestinal feeding tube (iT) The film is fixed with a suture coiled around the length of the tube. **b** detail of the DE segment. **c** Detail of the proximal end of the dOFD. Proximal opening of the gastric tube (G) to which negative pressure is applied. Proximal end of feeding tube (iT) with mandrin. Ventilation tube is blocked with a clamp (B)

**Fig. 2:**
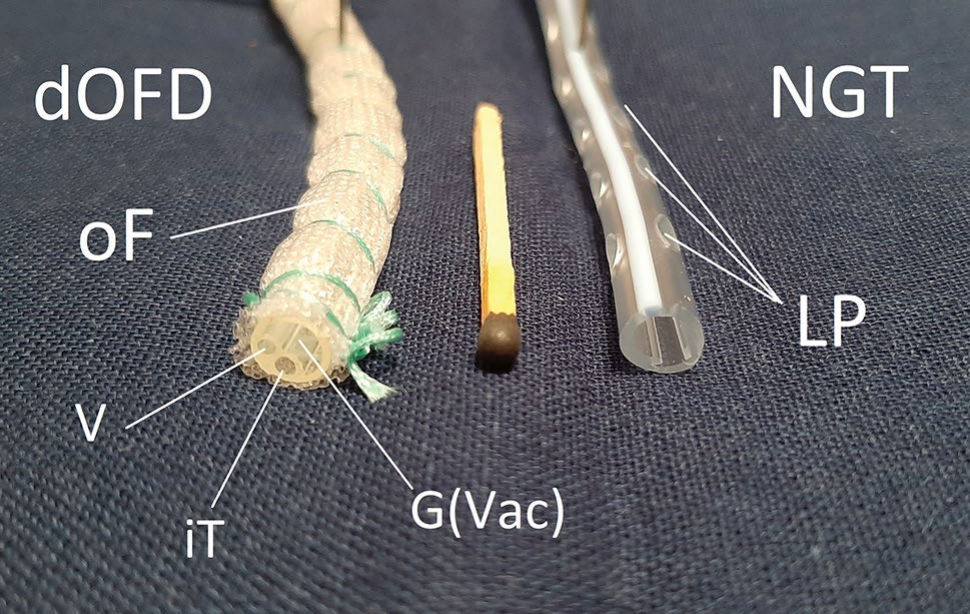
Illustration of the dOFD used and a NGT. The drains were cut open so that the transverse profile can be seen. The dOFD was wrapped with the open-pore double-layered film (oF). The oF has countless small pores on the surface, all of which are interconnected. The individual channels are integrated into one tube: Ventilation channel (V), this is blocked; intestinal feeding channel (iT); and the gastric channel (G(Vac)), to which the vacuum is applied and which is in suction contact with the oF. On the right, an 18 French NGT with large lateral perforations (LP) can be seen

For PARD, we used a dOFD as a NGT (Fig. 1). For construction, the distal end of the gastric channel of a triluminal tube (Freka®Trelumina, CH/Fr 16/9, 150 cm, Fresenius, Germany) is coated with a 25 cm long, 3–4 cm wide strip of a thin transparent open-pore double-layered film (Suprasorb® CNP Drainage film, Lohmann & Rauscher International GmbH, Germany) [5–7] (“How to make an dOFD” is demonstrated in the accompanying video). Originally, the drainage film was developed for use in intra-abdominal negative pressure therapy. The membrane consists of two perforated membranes with a small interspace in between. With negative pressure, liquids can be drained through the pores and along the interspace. The film is fixed with a suture to the tube. The ventilation channel of the tube is closed off. Because of the thinness of the film used as the open-pore drainage-element (DE), the small-bore dOFD has a diameter of only 6 mm (Fig. 2). Thus, the OFD can be inserted transnasally [5].

**Fig. 3:**
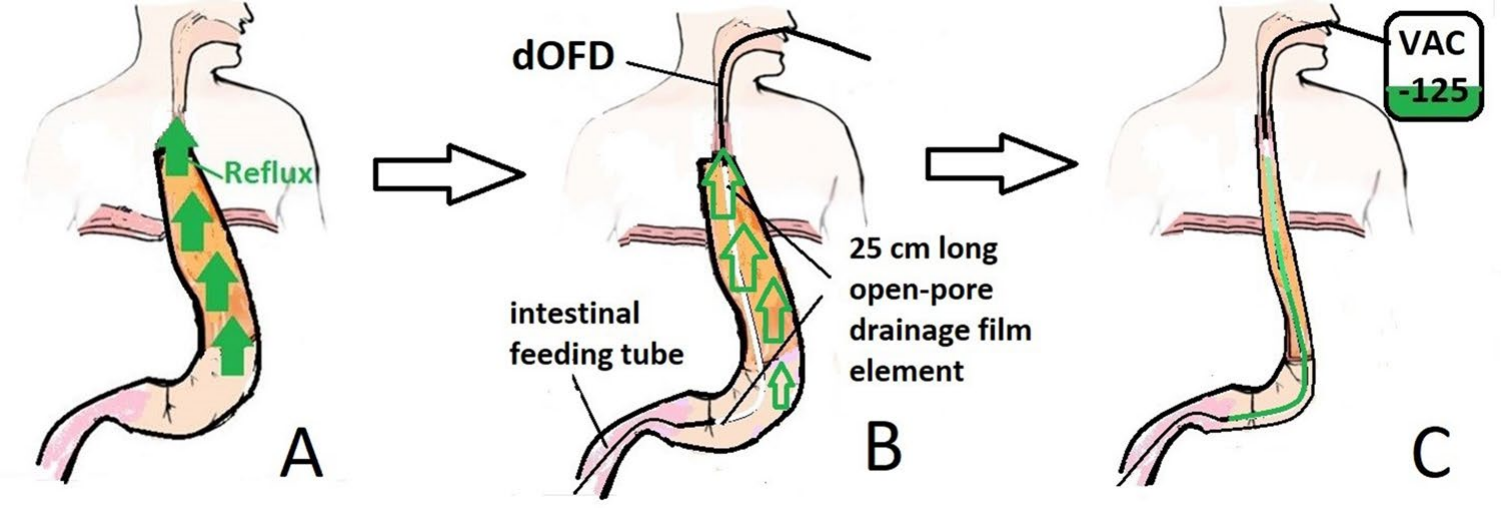
Schematic illustration of the PARD method. **A** Without a nasogastric tube postoperative reflux will flood the anastomotic region after ILE. **B** For PARD method, a thin double-lumen open-pore film drain (dOFD) is inserted through the nose. The 25 cm long open-pore drainage film element of the gastric tube is placed in the stomach. The intestinal feeding tube is directed into the duodenum. **C** With an electronic vacuum pump (VAC − 125) negative pressure of − 125 mmHg is applied to the gastric channel. Continuous negative pressure suction results in the permanent evacuation of the gastric conduit and decompression of the anastomotic region. Enteral feeding is possible via the feeding tube

The dOFD is inserted intraoperatively by endoscopic means directly after suturing the anastomosis. The DE is placed distal to the anastomosis, between the anastomosis and the pylorus. The intestinal feeding tube is directed into the duodenum. Correct placement of the DE is controlled endoscopically and from the open abdomen by the surgeon. The gastric channel of dOFD is then connected to an electronic vacuum pump (Activac, KCI USA Inc., San Antonio, Texas, United States) and negative pressure (− 125 mmHg, continuous suction) is applied (Fig. 3). To remove, the dOFD is simply pulled out of the nose. Depending on the findings in the endoscopic control, PARD can be terminated or continued with the insertion of a new dOFD.

The dOFD is a NGT with active suction. Since the use of a NGT an integral part of operative management after oesophagectomy, IBR approval was not needed. Written consent was obtained from all patients.

## Results

Between 11/2017 and 8/2020 PARD was used in all patients who underwent ILE in our clinic during this period. The group included 24 patients with ILE for cancer (18 male, 6 female, 53–77yo). 18/24 patients had undergone neoadjuvant oncological treatment before the operation. Individual patient data are shown in Table 1. The intrathoracic stapled anastomosis was located a median distance of 25 cm (15–30 cm) from the dental arch.

**Table 1:** Individual patient data

No.	Gender	Age (y)	TNM	Neo-adjuvant therapy	Insertion of OFD (n)	Healing of anastomosis	Duration of PARD (d)	At-risk anastomosis	Stenosis	Additional operations	Reflux on first post-op. Day (ml)
1	m	75	ypT3 yPN1	+	1	+	7	−			50
2	m	53	ypT3,ypN1	+	2	+	11	+			150
3	m	75	ypT2, pN0	+	2	+	10	+			375
4	f	53	ypT0, ypN0	+	2	+	8	−			100
5	m	63	pT1,pN0		1	+	4	−		+	250
6	f	72	pT1,pN0		2	+	8	−			450
7	m	62	ypT3ypN1	+	7	+	21	+	+		350
8	m	73	ypT2,pN0	+	6	+	19	+		+	350
9	f	79	ypT0 pN0	+	2	+	7	+			400
10	f	61	ypT3 pN1	+	2	+	10	−			500
11	m	67	ypT3 yPN2	+	2	+	8	+		+	250
12	m	64	ypT0 pN0	+	4	+	17	−		+	950
13	m	78	pT2 pN1		2	+	8	−			75
14	m	64	pT3 pN2		2	+	7	−			200
15	m	77	ypT3 pN1	+	4	+	10	+			480
16	f	66	pT1b pN0		2	+	10	+	+		670
17	m	59	ypT3 pN1	+	2	+	7	−			500
18	m	61	ypT0 pN0	+	1	+	4	−			900
19	f	75	ypT3 pN2	+	4	+	15	−		+	250
20	m	68	ypT3 pN0	+	5	+	15	+			100
21	m	68	ypT2 pN1	+	2	+	8	−		+	560
22	m	77	ypT3 pN0	+	5	+	13	+	+		350
23	m	72	ypT0 pN0	+	2	+	7	−			700
24	m	64	pT3 pN0		2	+	7	−			450
	18 m/6f	m = 68		*n* = 18	*m* = 2 (1–7)	*n* = 24 (100%)	*m* = 8 (4–21)	*n* = 10 (40%)	*n* = 3	*n* = 6	m = 360 (50–950)

Healing was observed in all the anastomoses (100%). No additional endoscopic interventions or operative revisions on the anastomosis were necessary.

The application of negative pressure to dOFD resulted in immediate drainage of PR. An endoscopic control was performed for the first time after a median of 4 (2–7) days. All endoscopies were performed with CO_2_ insufflation under short-acting anesthesia or sedation. Exchange of the dOFD with endoscopic evaluation of the wound situation was performed. PARD was terminated when stable anastomotic healing was confirmed endoscopically. All patients subsequently underwent further endoscopic examinations to confirm this.

**Fig. 4:**
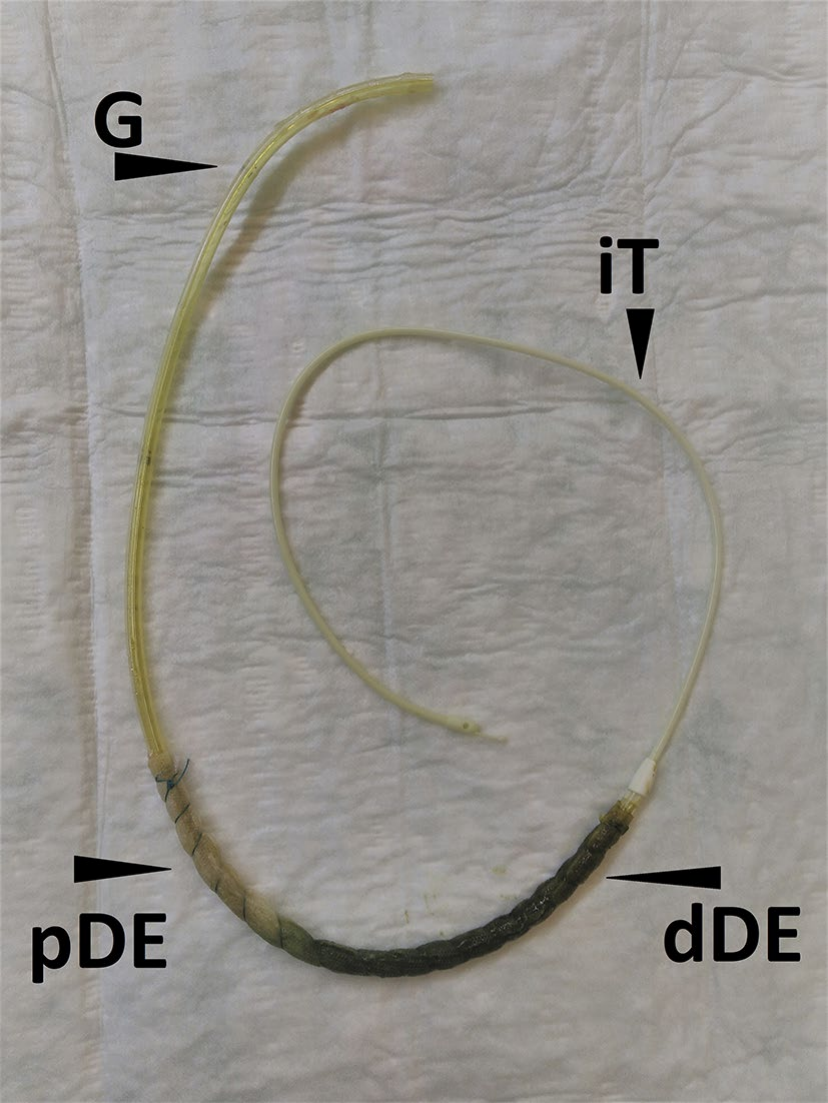
Demonstrates a dOFD after removal. Typically, the drainage-element is found to be saturated with green bile descending from distal drainage-element (dDE) to the proximal (pDE). The pores of the membrane in the pDE are blocked with swallowed bronchial mucus or gastric slime. In the dDE pores are patent and drainage is working

**Fig. 5:**
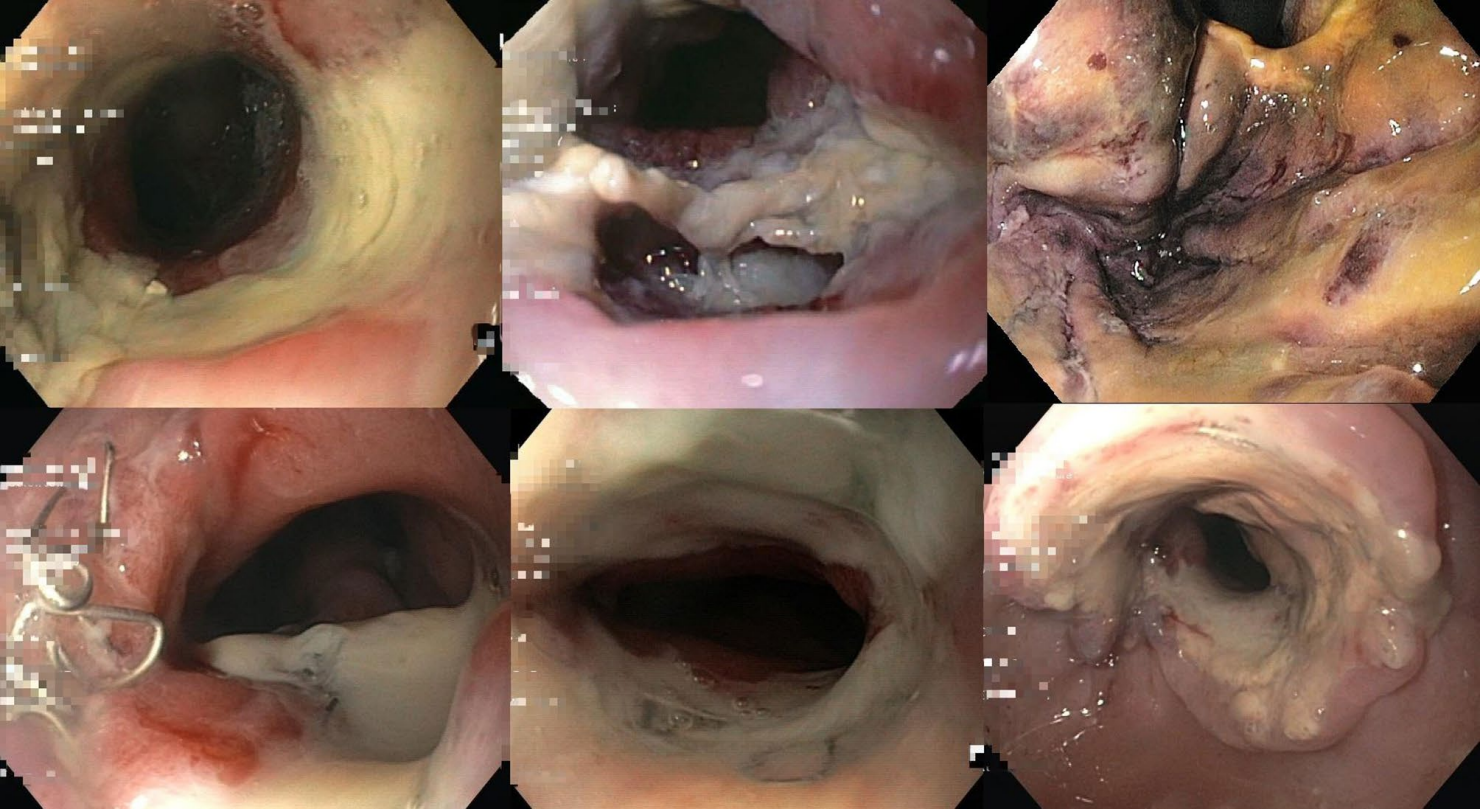
Endoscopic impressions of at-risk anastomoses (ARA) that healed using the PARD method

Endoscopy revealed complete elimination of PR and emptied gastric conduit in all patients. As seen in the removed dOFDs, the pores of the oral part of the DE, which was placed just below the anastomosis, were blocked due to ingested bronchial mucus and saliva. In the distal part of the DE, the pores were patent and functioning for the purposes of active negative pressure drainage. The DE was observed to be saturated with green bilious discolouration descending from distal to proximal (Fig. 4). In all endoscopic controls, the anastomoses were free of contamination with digestive secretions. The anastomotic tissue was found to be white in colour (Fig. 5).

In total, dOFDs were placed 56 times, with a median of 2 (1–7) per patient. PARD lasted for a median of 8 (4–21) days. The amount of reflux aspirated on the first postoperative day was *m* = 360 (50–950) ml/24 h. Enteral nutrition using the enteral feeding channel of dOFD was started gradually after operation. On two occasions it was observed that tube feeds were aspirated through the dOFD. This was a result of the feeding tube retracting into the stomach. The dOFD was then exchanged for a new one.

**Fig. 6:**
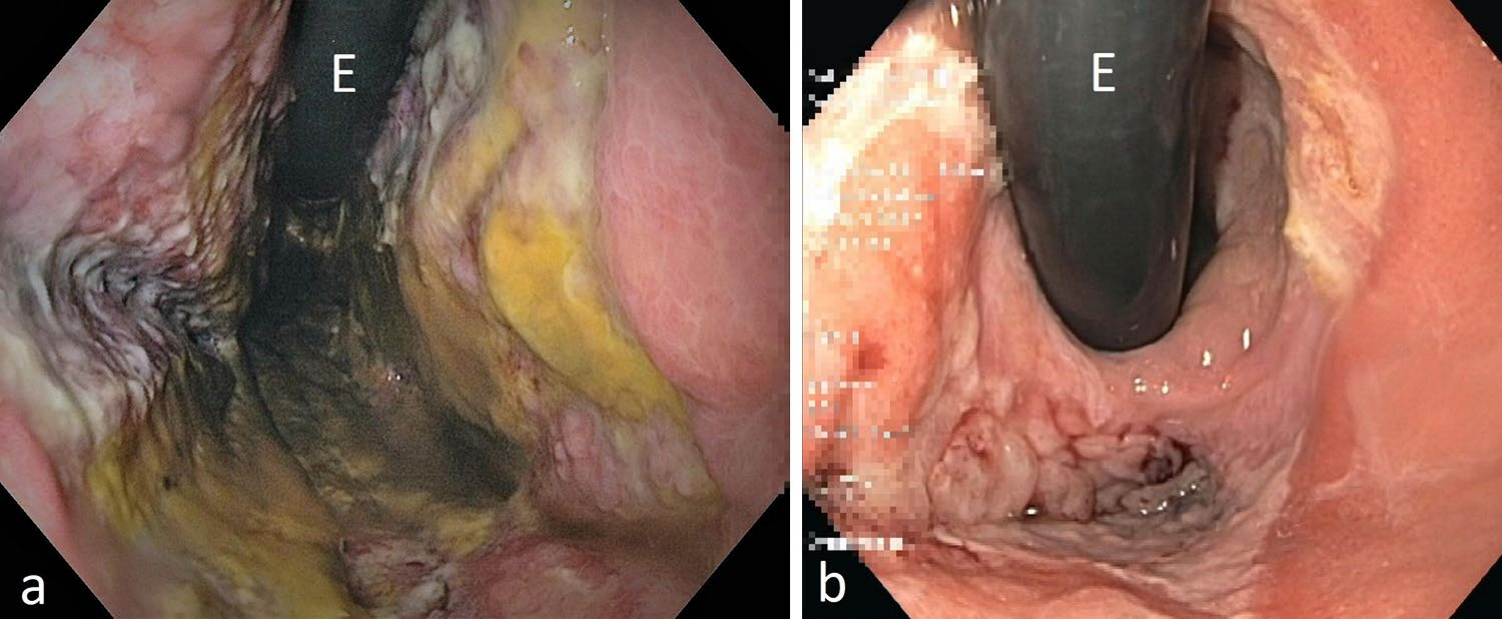
In patient No. 8 we found an incomplete ischaemia of the oral gastric conduit and anastomosis (**a**). During prolonged PARD for 19 day the necrosis was rejected, and secondary wound healing took place (**b**)

At the first endoscopic control, in 10/24 (40%) patients, problems with anastomotic healing were observed. The suspicious endoscopic findings were: visible suture material (clamps, sutures); widespread ulceration of the adjacent mucosa and epithelium, ischaemic adjacent tissue (Fig. 5). We defined anastomoses with these changes as “at risk anastomosis” (ARA) (Table 2). In these patients, PARD was continued until anastomotic healing was re-assessed and confirmed to be safe. For patients with ARA, the duration of PARD was 7–21 (median of 10.5) days. According to our protocol for intraluminal negative pressure therapy for anastomotic insufficiencies, a change of dOFD was performed twice weekly so that the therapy could be altered if necessary [8]. Four of the ten developed a short circular necrosis of the anastomosis. During PARD the anastomotic ischaemic tissue was rejected completely and replaced with granulation tissue. In long-term follow-up, three of them developed a mild yet concerning stenosis which was easily dilated with a balloon. In one patient we found an incomplete ischaemia of the oral gastric conduit and anastomosis. During prolonged PARD for 19 day the necrosis was rejected, and secondary wound healing took place (Fig. 6).

**Table 2:** Endoscopic criteria for an at-risk anastomosis defined

At-risk anastomosis (ARA)
Visable suture material (clamps, suture)
Broad ulceration of adjacent mucosa and epithelium
ischaemic adjacent anastomotic tissue

None of the ten patients with anastomotic problems developed signs of systemic sepsis during PARD. Local infection did not spread extraluminally in any of the patients. None developed mediastinitis. Even those patients with circular anastomotic necrosis or ischaemic gastric sleeve felt unaffected by the complications of internal wound healing. Full enteral nutrition was possible via the feeding tube.

For the comfort of our patients, they were allowed to take sips of water during prolonged PARD. This was also completely evacuated via the dOFD. No postoperative pulmonal aspiration occurred during PARD. No PR-induced oesophagitis was found. No other complications related to PARD were observed.

Six patients required additional operations (burst abdomen, bile duct injury, hepatobiliary fistula, gastroenterostomy, haemothorax, and chylothorax). Because of the additional surgeries, PARD was continued for longer periods in these patients. Two of them had an ARA.

One patient died 18 days after the operation due to a fulminant pulmonary infection; a relook of the anastomosis revealed no signs of pathological healing.

## Discussion

The intrathoracic anastomosis continues to be an anatomically vulnerable weak spot in the early postoperative period following ILE.

Several factors are known to influence anastomotic healing. Careful surgical techniques, good tissue perfusion and postoperative intermediate care management are prerequisites [2]. But even with the most accurate surgical technique, iatrogenic tissue damage resulting in reduced perfusion in the wound area is unavoidable. Furthermore, the intraluminal wound is exposed to reflux juice, which has digestive enzymatic effects on the anastomotic tissue. PR contains gastric, pancreatic, biliary and oral enzymatic secretions whose physiological purpose is digestion. PR induces oesophagitis and poses a risk of pulmonary aspiration [4].

Commonly used NGTs work passively by draining gases and fluids by means of gravity, capillary force or positive pressure. Their benefit is thought to be exerted through decompression of the stomach, thus helping to relieve tension in the anastomotic region. However, their utility is debated controversially [1].

PR flooding the anastomotic region is a frequently observed and well-known phenomenon after ILE. In numerous post-operative endoscopies after ILE, we have observed that even in the presence of a NGT, anastomoses were saturated with reflux [5]. The typical endoscopic evidence for this is an anastomosis imbibed with a greenish discolouration, as any endoscopist examining such patients postoperatively will be attentive to and confirm.

The main mode of action of the PARD method presented here is to prevent digestive reflux-related contamination of the anastomotic tissue in the early postoperative period. PARD using an dOFD eliminates PR permanently. The visible endoscopic evidence is that the anastomotic regions were free from bilious secretions and were whitish in colour. The method simultaneously results in complete decompression of the stomach and anastomotic region, and enteral feeding can also be undertaken.

When negative pressure is applied to common NGTs, their few lateral openings are sucked onto the gastric mucosa and blocked off. Open-pore drains used for endoscopic negative pressure therapy (ENPT) are coated with an open-pore material, either open-pore polyurethane foam or the thin double-layered open-pore film [8]. Even if some of the pores in these open-pore drainage materials are blocked, open-pore drains can continue to function to eliminate fluids. The open-pore characteristic enables permanently active guided drainage of fluids, even against gravity, when negative pressure is applied [9].

The dOFD used in this study is a special type of open-pore film drain (OFD) [5, 7]. The double-lumen design with its integrated feeding tube enables patients to be fed enterally while negative pressure suction is applied. Early enteral nutrition is considered a desirable post-operative intervention [10]. Especially when prolonged treatment is required, as is the case in our patients with an at-risk anastomosis, enteral nutrition has known advantages over a purely parenteral diet. PARD could also be undertaken using open-pore polyurethane foam drains (OPD) [8, 11–13]. Compared to OPDs, the advantage of an OFD is its lesser volume and the long length of the DE. Its small diameter of approximately 6 mm allows for insertion through the nose and easy handling, just like an NGT (Fig. 2) [5]

It could be said that an OFD is like an NGT, but with the additional benefit that suction can be applied.

The use of dOFD as a new tool for intraluminal ENPT (Endoscopic Negative Pressure Therapy) has been reported to be successful in the treatment of iatrogenic perforations of the oesophagus [14], Boerhaave`s syndrome [12] and anastomotic insufficiencies after sleeve gastrectomy [15]. In our clinical practice, the indications for dOFD are much broader. We use dOFDs to treat duodenal defects, preemptively after gastrectomy, as a supplement to ENPT or to break persistent reflux-induced aspiration in patients requiring intensive care. Single lumen OFDs have been used for several other endoscopic negative pressure therapy indications [7–9].

Correct placement of dOFD was ensured with intra-operative endoscopy [16]. The DE was placed in the gastric conduit just below the anastomosis to the pylorus since the goal was to eliminate reflux; the aim was not to apply suction to the anastomotic region. Principally, the initial intraoperative insertion of an OFD could be done even without endoscopy, with only digital control by the surgeon during open abdominal surgery.

In 40% of the patients, wound healing disturbances at the anastomosis were found in the first endoscopic control. With the protection of PARD, patients were clinically unaffected. Four of them developed ischaemic necrosis of the anastomoses and proximal gastric sleeve. Transmural defects were treated by continuing PARD with simultaneous enteral feeding only. All anastomoses healed; three developed short-stretch scarring anastomotic stenosis treated with balloon dilatation. The observed rate of ARA initially appears high [17], but correlates well with the current reported rate (24.5%) of anastomotic insufficiency [18]. In 2014, we presented our initial experience with ENPT for perforations and anastomotic insufficiencies in a patient cohort of 35 patients; the incidence for esophageal anastomotic insufficiency in our cohort at that time was 17% [19].

We assume that at least some of the ten patients with ARA would have developed manifest severe anastomotic insufficiency without PARD.

A similar observation was made by Neumann et al. They demonstrated successful pre-emptive intraluminal ENPT with OPD-devices in eight patients with postoperative ischaemic oesophageal anastomosis [20]. A polyurethane sponge-based OPD was placed intraluminally directly onto the anastomosis. Two of the eight patients developed an anastomotic leak which was treated with ENPT only. All the anastomoses healed.

Gubler et al. reported intraoperative pre-emptive intraluminal ENPT in a first clinical series of 19 patients with 20 anastomoses [21]. They inserted a commercial OPD (EsoSponge, B.Braun, Melsungen, Germany) intraluminally onto the anastomotic region directly after oesophagectomy. The healing rate of anastomosis was 95%; one patient developed a small anastomotic leak treated solely with ENPT. Recently, the working group has launched an international multicentre study to evaluate pre-emptive intraluminal ENPT in the oesophagus [2].

The pre-emptive use of intraluminal negative pressure is supported by a study from Scott et al. They demonstrated the use of prophylactic intraoperative intraluminal ENPT in a series of pigs with ILE. After creating the anastomosis, a transmural defect was left which was covered with an intraluminal OPD. Intraluminal ENPT was started intraoperatively. All defects were found to be closed after a treatment period of 5 days [22].

These studies suggests that intraluminal ENPT may have a favorable impact on anastomotic healing in the oesophagus. The two mechanisms of action of intraluminal ENPT are, firstly, sealing the anastomosis, and secondly, drainage of secretions [8, 11]. The importance of active drainage of bile secretions was demonstrated recently by the treatment of duodenal defects with ENPT [9, 23].

In this study, our aim was not to cover the anastomotic area with negative pressure drainage, which would be technically very easy with a longer DE. Our goal was to use the PARD method to keep the anastomosis completely and permanently free of digestive secretions. This single measure seems to have a high protective effect on anastomotic healing, even though there are alarming disturbances of wound healing at the anastomosis. This protective effect allows anastomotic healing to occur unperturbed. We conclude from our study that the drainage effect is of particular importance. We consider this observation to be the most significant result of our work. According to our preliminary experience, PARD could be a suitable method to increase patient safety for ILE. It follows that PARD could be a protective method in the sense of real prophylaxis, which supports anastomotic healing on the one hand and, on the other, prevents the serious consequences of a wound healing disorder at the anastomosis, including the complicating and life-threatening consequences of anastomotic insufficiency [24]. In our clinic PARD has been introduced as a standard procedure for ILE.

We would like to mention potential limitations of the study. Firstly, the study has a small sample size. However, it is currently the largest study describing the PARD method for ILE with simultaneous enteral nutrition using the thin dOFD. Our observations and results should be confirmed by other surgical-endoscopic centers. This should be easily reproducible based on the detailed presentation and ease of use of the method. Secondly, this was a retrospective observational study on PARD using a single type of dOFD. The advantages of dOFD are its thin diameter, easy insertion into the drain, and the possibility of simultaneous nutrition. PARD can also be carried out using a single-lumen OFD and OPD or a double-lumen OPD [13, 25]. Prospective studies comparing these types of open-pore drains with common NGTs would be desirable. The issue of drain placement (transpyloric duodenal/gastric) may also be of interest. It should be mentioned that the number of endoscopic procedures is increasing with the inherent risks and use of resources. Thirdly, our study suggested that the drainage of PR alone conferred a strong protective effect. Negative pressure applied directly to the anastomotic region could increase the pre-emptive action. Future pre-emptive negative pressure studies using a PARD arm should aim to answer this question. Finally, we used negative pressure of − 125 mmHg, which is our standard setting for ENPT in upper and lower gastrointestinal tract. There is no evidence on this pertaining to PARD, and it is conceivable that a lower negative pressure may be sufficient [26].

## Conclusion

Intraoperative onset PARD with a dOFD for ILE is a simple endoscopic method to prevent PR in the early vulnerable post-operative period at the anastomoses. Negative pressure applied to the dOFD (a nasogastric tube (NGT)) enables enteral nutrition to be delivered simultaneously with permanent evacuation and decompression. PARD seems to have a strong protective effect on anastomotic healing and may reduce the rate of anastomotic insufficiency.

## Supplementary Information

Below is the link to the electronic supplementary material.

Supplementary file1 (MP4 355111 kb)

## Abbreviations

ARA: At-risk anastomosis
DE: Drainage element
dOFD: Double-lumen open-pore film drainage
ENPT: Endoscopic negative pressure therapy
ILE: Ivor-Lewis oesophagectomy
NGT: Nasogastric tube
OFD: Open-pore film drain
OPD: Open-pore polyurethane foam drain
PARD: Pre-emptive active reflux drainage
PR: Postoperative reflux

## References

[1] Weijs TJ, Kumagai K, Berkelmans GH, Nieuwenhuijzen GA, Nilsson M, Luyer MD (2017). Nasogastric decompression following esophagectomy: a systematic literature review and meta-analysis. Dis Esophagus.

[2] Vetter D, Gutschow CA (2020). Strategies to prevent anastomotic leakage after esophagectomy and gastric conduit reconstruction. Langenbecks Arch Surg.

[3] Grigor E, Kaaki S, Fergusson DA, Maziak DE, Seely A (2021). Interventions to prevent anastomotic leak after esophageal surgery: a systematic review and meta-analysis. BMC Surg.

[4] Usui H, Fukaya M, Itatsu K, Miyata K, Miyahara R, Funasaka K, Nagino M (2018). The impact of the location of esophagogastrostomy on acid and duodenogastroesophageal reflux after transthoracic esophagectomy with gastric tube reconstruction and intrathoracic esophagogastrostomy. World J Surg.

[5] Loske G, Schorsch T, Müller CT (2017). Prevention of reflux after esophagectomy with endoscopic negative pressure therapy using a new double-lumen open-pore film drainage with an intestinal feeding tube. Endoscopy.

[6] Loske G (2019). Endoscopic negative pressure therapy of the upper gastrointestinal tract. Chirurg.

[7] Loske G, Schorsch T, Rucktaeschel F, Schulze W, Riefel B, van Ackeren V, Mueller CT (2018). Open-pore film drainage (OFD): a new multipurpose tool for endoscopic negative pressure therapy (ENPT). Endosc Int open.

[8] Loske G, Müller CT (2019). Tips and tricks for endoscopic negative pressure therapy. Chirurg.

[9] Loske G, Rucktaeschel F, Schorsch T, Moenkemueller K, Mueller CT (2019). Endoscopic negative pressure therapy (ENPT) for duodenal leakage - novel repair technique using open-pore film (OFD) and polyurethane-foam drainages (OPD). Endosc Int Open.

[10] Weimann A, Braga M, Carli F, Higashiguchi T, Hübner M, Klek S, Laviano A, Ljungqvist O, Lobo DN, Martindale R, Waitzberg DL, Bischoff SC, Singer P (2017). ESPEN guideline: clinical nutrition in surgery. Clin Nutr.

[11] Loske G, Schorsch T, Müller C (2011). Intraluminal and intracavitary vacuum therapy for esophageal leakage: a new endoscopic minimally invasive approach. Endoscopy.

[12] Loske G, Albers K, Mueller CT (2021). Endoscopic negative pressure therapy (ENPT) of a spontaneous oesophageal rupture (Boerhaave’s syndrome) with peritonitis—a new treatment option. Innov Surg Sci.

[13] Loske G, Aumiller J, Rucktäschel F, Schorsch T (2016). Spontaneous perforation of an intramural esophageal pseudodiverticulosis treated with intraluminal endoscopic vacuum therapy using a double-lumen vacuum drainage with intestinal feeding tube. Endoscopy.

[14] Wichmann D, Stüker D, Schempf U, Werner CR, Steger V, Königsrainer A, Schweizer U, Archid R (2020). Endoscopic negative pressure therapy with open-pore film drainage and open-pore polyurethane sponge drainage for iatrogenic perforation of the esophagus. Endoscopy.

[15] Archid R, Wichmann D, Klingert W, Nadiradze G, Hönes F, Archid N, Othman AE, Ahmad S, Königsrainer A, Lange J (2020). Endoscopic vacuum therapy for staple line leaks after sleeve gastrectomy. Obes Surg.

[16] Park JH, Jeong SH, Lee YJ, Kim TH, Kim JM, Kim DH, Kwag SJ, Kim JY, Park T, Jeong CY, Ju YT, Jung EJ, Hong SC (2020). Safety and efficacy of post-anastomotic intraoperative endoscopy to avoid early anastomotic complications during gastrectomy for gastric cancer. Surg Endosc.

[17] Schaible A, Ulrich A, Hinz U, Büchler MW, Sauer P (2016). Role of endoscopy to predict a leak after esophagectomy. Langenbecks Arch Surg.

[18] Liesenfeld LF, Sauer P, Diener MK, Hinz U, Schmidt T, Müller-Stich BP, Hackert T, Büchler MW, Schaible A (2020). Prognostic value of inflammatory markers for detecting anastomotic leakage after esophageal resection. BMC Surg.

[19] Schorsch T., Müller C., Loske G. (2014) Endoskopische Vakuumtherapie von Perforationen und Anastomoseninsuffizienzen des Ösophagus [Endoscopic vacuum therapy of perforations and anastomotic insufficiency of the esophagus] Chirurg. 85(12):1081–1093 10.1007/s00104-014-2764-4 24920346

[20] Neumann PA, Mennigen R, Palmes D, Senninger N, Vowinkel T, Laukoetter MG (2017). Pre-emptive endoscopic vacuum therapy for treatment of anastomotic ischemia after esophageal resections. Endoscopy.

[21] Gubler C, Vetter D, Schmidt HM, Müller PC, Morell B, Raptis D, Gutschow CA (2019). Preemptive endoluminal vacuum therapy to reduce anastomotic leakage after esophagectomy: a game-changing approach?. Dis Esophagus.

[22] Scott RB, Ritter LA, Shada AL, Feldman SH, Kleiner DE (2017). Endoluminal vacuum therapy for ivor lewis anastomotic leaks: a pilot study in a swine model. Clin Transl Sci.

[23] de Moura DTH, do Monte ES, Hathorn KE, de Medeiros FS, Thompson CC, de Moura ECH (2021). Modified endoscopic vacuum therapy in the management of a duodenal transmural defect. Endoscopy.

[24] Schniewind B, Schafmayer C, Voehrs G, Egberts J, von Schoenfels W, Rose T, Kurdow R, Arlt A, Ellrichmann M, Jürgensen C, Schreiber S, Becker T, Hampe J (2013). Endoscopic endoluminal vacuum therapy is superior to other regimens in managing anastomotic leakage after esophagectomy: a comparative retrospective study. Surg Endosc.

[25] Archid R, Bazerbachi F, Thomas MC, Königsrainer A, Wichmann D (2020). Endoscopic negative pressure therapy for upper gastrointestinal leaks: description of a fashioned device allowing simultaneous enteral feeding. VideoGIE.

[26] Jung CFM, Müller-Dornieden A, Gaedcke J (2020). Impact of endoscopic vacuum therapy with low negative pressure for esophageal perforations and postoperative anastomotic esophageal leaks. Digestion.

